# Gut Microbiota Modulation and Fecal Transplantation: An Overview on Innovative Strategies for Hepatic Encephalopathy Treatment

**DOI:** 10.3390/jcm10020330

**Published:** 2021-01-18

**Authors:** Ramzi Hassouneh, Jasmohan S. Bajaj

**Affiliations:** 1 Department of Internal Medicine, Virginia Commonwealth University Medical Center, Richmond, VA 23298, USA; Ramzi.Hassouneh@vcuhealth.org; 2 Division of Gastroenterology, Hepatology and Nutrition Virginia Commonwealth University and Central Virginia Veterans Healthcare System, 1201 Broad Rock Blvd, Richmond, VA 23249, USA

**Keywords:** cirrhosis, bile acids, antibiotic resistance, enema, capsules, hospitalizations, cognitive function

## Abstract

**Simple Summary:**

Treatment of advanced liver disease and its complications continue to be a challenge due to the complexity of this illness. In recent years, the gut microbiome has been recognized to play a beneficial role in our health. Studies have shown that overgrowth of harmful organisms in the gut can contribute to worsened outcomes in liver disease. Fecal microbiota transplant (FMT) is an approved and effective treatment in other gastrointestinal conditions. FMT involves the administration of a solution of a fecal suspension from a healthy donor into the intestinal tract of a recipient. This has led researchers to attempt this treatment in liver disease. There have now been small clinical trials showing that FMT is safe and could be effective in improving outcomes in advanced liver disease. There remain several questions to be answered before FMT is implanted in clinical practice, including the best route to administer this treatment, how many doses are needed to achieve a therapeutic response, and how long we need to wait between treatments. In this review paper, we explore the role of the gut microbiome in the human body with emphasis on the gastrointestinal system, how it changes in liver disease, and how we can improve it with fecal microbiota transplant.

**Abstract:**

Hepatic encephalopathy (HE) is a major complication of cirrhosis, which is associated with gut microbial composition and functional alterations. Current treatments largely focus on gut microbiota using lactulose, rifaximin and other agents. However, despite these treatments, patients with HE have a high rate of readmission, morbidity and cognitive impairment. Fecal microbiota transplant (FMT) involves introduction of a donor microbiota into a recipient and is currently mainly used for recurrent *C. difficile* infection (rCDI). The role of FMT in cirrhosis and HE is evolving. There have been two randomized clinical trials (RCT) and several case reports/series in cirrhosis. Both RCTs were safety-focused phase 1 trials. One involved pre-FMT antibiotics and FMT enema versus standard of care, while the other involved 15 FMT capsules versus placebo without pre-FMT antibiotics. There was evidence of safety in both trials and the FMT group demonstrated reduction in hospitalizations compared to the non-FMT group. Changes in microbial function centered around short-chain fatty acids, bile acids and brain function showed improvement in the FMT groups. Long-term follow-up demonstrated continued safety and reduction in the antibiotic-resistance gene carriage. However, larger trials of FMT in HE are needed that can refine the dose, duration and route of FMT administration.

## 1. Introduction

Liver cirrhosis has a multitude of debilitating complications including ascites, bleeding and hepatic encephalopathy (HE). HE is a major neurologic complication of cirrhosis and is estimated to affect between 30 to 70% of patients with cirrhosis [1,2]. It often requires hospitalization, which has a significant economic impact. Studies have shown that the yearly total cost for patients with a primary diagnosis of HE is estimated at USD 620 million in the United States [3]. Cognitive impairment experienced by patients with HE can range from covert (minimal) to overt [2]. Covert HE can be subtle in nature and requires the use of specialized tests for detection. In contrast, overt HE is characterized by behavioral changes, confusion and lethargy [2]. Regardless of type, HE portends a poor prognosis and is associated with increased morbidity [1]. It not only negatively impacts the patient’s quality of life but also places a heavy burden on family and caregivers. Moreover, individuals who experience HE have a high chance of recurrence [1,2].

The exact pathogenesis for the development of HE is yet to be determined. Certain mechanisms have been implicated and have been recognized for years including increased production of neurotoxins, impairment of neurotransmission, systemic inflammation, alteration of the blood–brain barrier and alterations in energy metabolism [4,5]. It is clear that no single entity is solely responsible for HE, and it is a synergistic effect of multiple mechanisms that lead to this illness. In recent years, it has been increasingly recognized that the gut microbiota plays a large role in the development of HE [6,7,8]. This is most evident by the beneficial role of antibiotics in the treatment in HE, which typically decrease the intestinal population of urease-producing microbes [6,7,8]. This suggests that HE is a disease of the gut–liver–brain axis whereby dysregulation in gut microbiota can lead to many downstream effects including bacterial translocation and toxin production subsequently causing systemic and neurological inflammation [6,7,8]. This has led to the evaluation of gut microbiota as a potential therapeutic target, particularly with fecal microbiota transplantation (FMT), and early studies have shown promising results [9].

## 2. Composition of Gut Microbiota

The number of microbes in the gut vastly outnumber the number of somatic cells in the human body [10,11,12,13]. There are more than a trillion unique microbial species found throughout the gastrointestinal tract [10,11,12,13]. At birth, the gut is relatively sterile, and microorganisms start colonizing after feeding [10,11,12,13]. The composition of the gut microbiota differs among individuals and is altered during times of illness and with dietary changes. The majority of organisms tend to be anaerobic bacteria with the greatest representation by species that belong to the phyla Bacteroidetes and Firmicutes [10,11,12,13,14]. Additionally, the population of microbes increases distally with the greatest amount and diversity of microbes seen in the distal small intestine and colon [10,11,12,13,14]. The stomach and proximal intestine contain a small number of organisms (10^2^), which are made up of a combination of Gram-positive and Gram-negative bacteria as well as fungal species such as *Lactobacillus*, *Streptococcus*, *Helicobacter* and *Candida* [10,11,12,13,14]. The distal intestine and colon contain high levels (10^6–12^) of predominantly anaerobic bacteria including Firmicutes and Bacteroidetes phyla as well as *Clostridium* species [10,11,12,13,14].

## 3. Gut Microbiota Function

The gut microbiota is involved in several normal physiological processes. It facilitates digestion by extracting and absorbing carbohydrates, amino acids, lipids, vitamins and bile acids. It also inhibits growth of invasive microorganisms by preferentially utilizing available resources, by producing anti-bacterial molecules and by contributing to the maintenance of the intestinal immune system. This interplay between the human body and the gut microbiota is a beneficial synergistic relationship that promotes the health of both parties and maintains homeostasis. This state of homeostasis is referred to as eubiosis.

As alluded to earlier, illness can significantly alter the composition of the gut microbiota. This alteration of its composition is known as dysbiosis, which is defined as a pathological condition that disturbs the state of homeostasis. The microbiota responsible for maintaining homeostasis is mainly the Firmicutes organisms, which have been shown to be severely decreased in liver disease [15,16]. Dysbiosis is heavily implicated in cirrhosis and HE. The etiology for dysbiosis in cirrhosis is presumed to involve reduced levels of bile acids and short chain fatty acids (SCFA), small intestinal bacterial overgrowth (SIBO), and immune dysregulation [17,18,19,20,21,22,23,24,25]. Bile acids are thought to be protective against dysbiosis, as they are involved in the lysis of pathogens [19,20,21,22]. Bile acids have also been shown to regulate innate and adaptive immune inflammatory signaling in the gut by modulating the differentiation of Th17 and T_reg_ cells [23]. Patients with cirrhosis produce lower levels of bile acids due to poor biosynthetic function and impaired intestinal secretion [19,20,21,22]. SIBO is commonly seen in cirrhosis; this increases the quantity of pathological organisms and their metabolites, which has several downstream effects including changes to intestinal permeability [24,25,26]. SCFA are by-products of gut bacterial carbohydrate metabolism, and they have been shown to be integral in maintaining luminal pH, intestinal motility and enterocyte structure [23]. SCFA also regulate immune response in gut lymphoid tissue by inhibiting macrophages, dendritic cells and inflammatory cytokines [23]. Furthermore, gut lymphoid tissue express pattern recognition receptors such as toll-like receptors, which recognize commensal bacterial antigens, and this leads to a cascade of signals that ultimately lead to the differentiation of naïve T cells [23]. The absence or reduction in these commensal bacterial antigens hinders the proliferation and differentiation of gut lymphoid population [23]. In summation, dysbiosis in cirrhosis promotes a pro-inflammatory and immunosuppressed state.

## 4. Changes to Gut Microbiota in Cirrhosis and Hepatic Encephalopathy

Many studies have shown that there is a significant difference between the stool microbiota composition in healthy individuals compared to individuals with cirrhosis. Beneficial organisms that are normal residents of the gut and contribute to the state of homeostasis are referred to as autochthonous organisms. Studies have shown that in cirrhosis there is a reduction in autochthonous organisms such as Bacteroidetes and an increase in harmful organisms such as *Enterobacteriaceae* [27,28]. The data from human studies on gut microbiota in cirrhosis are summarized in Table 1. On the one hand, autochthonous organisms have several beneficial roles including the production of bile acids and SCFAs (e.g., butyrate). On the other hand, pathogenic organisms such as *Enterobacteriaceae* produce endotoxins and lipopolysaccharides, which promote inflammation. The outgrowth of these pathogenic organisms results in the loss of beneficial autologous species, resulting in reduced levels of bile acids and SCFAs and serves to increase intestinal inflammation and permeability, which in turn allows bacterial translocation and systemic inflammation. Interestingly, several studies have shown that not only does the microbiota detected in stool change but also the microbiota detected in the oral cavity. These organisms such as *Streptococcaeae* and *Veillonellaceae* are seen more abundantly in the oral cavity in individuals with cirrhosis and are correlated with a worse disease prognosis. In addition, studies have shown that dysbiosis in cirrhosis is not only limited to stool and saliva but also in the colonic and duodenal mucosa as well as in the liver and ascitic fluid. This further supports the principle of cirrhosis and its complications being a systemic disease with multiple pathophysiologic mechanisms in play.

The changes in composition of the microbiota in cirrhosis are important because of their effect on metabolite and byproduct levels and their ability to affect the body. Many metabolites require microorganisms for their production including bile acids, dimethylamine/trimethylamines and hippurate [17,18,19,20,21,22,35,46]. As described earlier, endotoxins and lipopolysaccharides increase in cirrhosis when the population of deleterious organisms increase. These perturbations are further exacerbated by underlying portal hypertension in cirrhosis, which leads to bypassing of the reticuloendothelial system and delivery of these metabolites to the systemic circulation. Autochthonous organisms such as Clostridia are responsible for converting primary bile acids to secondary bile acids, which subsequently contributes to fecal bile acid concentrations [17,18,19,20,21,22,35]. Reductions in fecal bile acid was observed in patients with cirrhosis with a concomitant increase in serum bile acid. Methylamines are associated with atherosclerosis and cardiovascular disease and is now recognized to play a role in liver disease [47,48,49]. Alteration of levels of bacterial-derived methylamines was observed in individuals with cirrhosis [47,48,49]. Hippurate is a product of bacterial metabolism of dietary polyphenols, and low levels of hippurate have been observed in individuals with liver failure and are linked to the degree of hepatocellular reserve [25,50]. Changes to relative abundance of *Bacteroidaceae* in cirrhosis is thought to perhaps contribute to reductions in hippurate levels [36].

All these changes to microbiota population and function have been invariably linked to complications of cirrhosis including HE. For instance, patients with HE have an increased population of *Veillonellaceae*, which was associated with increased levels of IL-6, TNF-a, IL-2 and IL-13 [51]. Elevations in pro-inflammatory cytokines are thought to have contributed to poorer cognition in patients with HE [6]. HE is associated with over-abundance of ammonia partly due to urease-producing organisms. Individuals with HE and poor cognition were found to have higher population of urease-producing Proteobacteria [6]. Another study showed that *Blautia*, *Fecalibacterium*, *Roseburia* and *Dorea* were associated with good cognition, while pathological organisms such as Enterococcus was linked to poor cognition [33]. Furthermore, an over-abundance of ammoniagenic oral organisms such as *Streptocoocus* species were found in cirrhotic patients with HE [34]. Endotoxin-producing organisms were also found in higher abundance in the oral microbiota in patients with HE, which is correlated with a pro-inflammatory state with increased levels of IL-1B, IL-6 and IgA [34]. Magnetic resonance imaging (MRI) has been utilized in some studies to evaluate the link between the composition of gut microbiota and neuronal function [41]. By using brain MRI, patients with higher levels of Enterobacteriaceae were found to be have astrocytic changes typically seen in hyperammonemic states [41]. Furthermore, patients with higher levels of *Porphyromonadaceae* were found to have non-hyperammonemic neuronal changes [41].

As described earlier, SIBO is commonly associated with cirrhosis, affecting up to 59% of these patients [52,53,54,55,56,57,58,59,60,61,62,63]. The gold standard diagnostic method is a demonstration of greater than 10^5^ colony forming units per millimeter in the proximal jejunum via aspiration during endoscopic examination. SIBO typically shows an over-abundance of gram-negative bacteria including *Escherichia coli* and *Klebsiella pneumoniae* [52,53,54,55,56,57,58,59,60,61,62,63]. These organisms are linked to the development of decompensated cirrhosis and the development of HE due to their ability to translocate [52,53,54,55,56,57,58,59,60,61,62,63]. One meta-analysis demonstrated that the prevalence of SIBO in cirrhosis is significantly greater than its prevalence in otherwise healthy individuals [58]. In this study, the presence of SIBO in cirrhotic patients was correlated with an increased probability of HE compared to those without SIBO [58]. SIBO alone, therefore, does not predispose to liver disease, but it does predispose to worse outcomes when it coexists with systemic inflammation, immunodeficiency, increased intestinal permeability and decreased intestinal motility.

## 5. Modulation of Gut Microbiota in Hepatic Encephalopathy

The first-line therapy for HE is to target the precipitating episode, which can range from bleeding, dehydration, constipation to infection. Other therapies can be started once the precipitating episode is identified and treated. The most common therapies for HE include non-absorbable disaccharides such as lactulose and lactitol as well as antibiotics such as rifaximin. These therapies work by decreasing serum ammonia levels by accelerating intestinal transit and by modifying intestinal bacterial metabolism and abundance. As the influence of gut microbiota composition on cirrhosis and HE has been clearly demonstrated by many studies, the next natural step is to therapeutically target the gut–liver axis. This has been done with dietary changes, prebiotics, probiotics and more recently with fecal microbiota transplantation (FMT).

Diets rich in plant protein, fermented milk products, and vegetables have shown to be beneficial to the gut microbiota. Plant protein has been linked to greater abundance of autochthonous organisms and reduction in pro-inflammatory organisms [64]. We demonstrated that diet not only affects gut microbiota but also modulates hospitalization risk [65]. This was done by comparing decompensated and compensated American patients with cirrhosis to matched cohorts in Turkey. Turkish patients were found to have significantly higher microbial diversity with higher intake of vegetables, chocolate, coffee, tea and fermented milk intake predicting a higher microbial diversity. Furthermore, the Turkish cohort had a lower risk of 90-day hospitalizations.

Administration of probiotics and prebiotics is another technique to modulate the gut microbiota. The purported mechanism of probiotics is to directly increase the population of beneficial bacteria. The data on probiotic therapy are conflicting, especially for treating gastrointestinal pathologies other than liver disease; however, one study showed benefit in cirrhosis complicated by HE [66,67]. Saab et al. demonstrated that probiotics compared to placebo decreased hospitalization rates in patients with cirrhosis and HE and prevented progression to overt HE in patients with underlying covert HE [68]. Prebiotics are typically non-digestible fiber compounds that feed beneficial bacteria in the digestive system. Prebiotics have been shown to promote the growth of organisms that produce SCFAs and thus preventing intestinal permeability [67].

## 6. Fecal Microbiota Transplantation in Hepatic Encephalopathy

FMT involves the administration of a solution of a fecal suspension from a donor into the intestinal tract of a recipient. This can be administered in several formulations including enema, via colonoscopy, or in capsular form. This therapy serves to directly change the gut microbiota composition. Previous studies have shown that FMT is effective in conditions associated with dysbiosis particularly recurrent *Clostridoides difficile* and ulcerative colitis [69,70]. As repeatedly demonstrated, cirrhosis and, in particular, HE is associated with dysbiosis. Reversing dysbiosis and restoring eubiosis can potentially reduce systemic inflammation, preserve gut membrane integrity, prevent bacterial translocation and maintain production of bile acids.

A case report by Kao et al. was the first attempt at treating HE with FMT [71]. Cognition was assessed with the inhibitory control test and the Stroop test, and they showed that the patient’s cognition improved with consecutive FMT until it stabilized by the fourth week after a total of three FMTs. The patient’s cognition reverted to baseline after 14 weeks after withdrawal of FMTs. Interestingly, *Lachnospiraceae*, which is associated with better cognition, was found to be in reduced quantities in this patient.

The aforementioned results were promising and lead to the first randomized control trial studying the efficacy of FMT in HE, which was conducted by our group. In this study, a single stool specimen was used for the experimental group by identifying a donor with the highest relative abundance of *Lachnospiraceae* and *Ruminococcaceae* [72]. A total of 20 cirrhotic patients with recurrent HE (defined as two or more episodes) were enrolled and randomly assigned to the standard of care and to the FMT group. Both groups were to continue their lactulose and rifaximin. Prior to receiving the FMT enema, the experimental group received a five-day treatment of antibiotics to increase the success of donor bacterial colonization. The FMT with antibiotic pretreatment was well tolerated, and the patients were followed for up to 150 days. There was a significant reduction in serious adverse events in the experimental group compared to the control group. Five patients in the control group had a recurrent episode of HE, whereas no patient in the FMT group developed further HE. The psychometric hepatic encephalopathy score (PHES) and EncephalApp Stroop (EAS) were used to assess cognitive ability. These cognitive assessments showed that there was a significant improvement in cognition in the FMT group at day 20 compared to baseline.

A follow-up study was performed to demonstrate the long-term safety of FMT. The study was carried out in a similar fashion to our previous study; however, all participants received proton pump inhibitors in addition to lactulose and rifaximin. The participants were followed for 12 months, and there were significantly fewer hospitalizations and HE episodes in the FMT group compared to the control group [73]. Furthermore, cognition measured by PHES and EAS was found to be significantly better in the experimental group compared to their control counterparts. Similar to the case report by Kao et al., the abundance of the autochthonous organisms *Lachnospiraceae* and *Ruminococcaeae* was not statistically different between the two groups despite the FMT donor’s microbiome being rich in these organisms. We concluded that FMT is safe for long-term use; however, this study was limited by a small sample size. This was then followed by a phase 1, randomized, placebo-controlled trial to determine the safety of capsular FMT. The groups were followed for five months after administration of FMT capsules obtained from a single donor with the highest relative abundance of *Lachnospiraceae* and *Ruminococcaceae*. Only one serious adverse event was reported in the FMT group as opposed to 11 in the control group, suggesting that FMT capsules are well tolerated and safe [74]. Patients who received FMT capsules had improved cognition when assessed with EAS; however, PHES did not improve. The participants had their microbiomes analyzed, and the FMT group had increases in duodenal *Ruminococcaeae* and *Bifidobacteriaceae* and decreases in *Streptococcaceae* and *Veillonellaceae*. Moreover, there were reductions in sigmoid and stool populations of *Veillonellaceae*. As described earlier, *Streptococcaceae* is an ammionagenic organism and is linked with development of HE. There was a non-significant trend toward fewer hospitalizations in the FMT group. Although the sample size was small, this was one of the first studies to demonstrate that FMT capsules can decrease relative abundance of microorganisms associated with the progression of cirrhosis. A case series published by Mehta et al. performed FMT via colonoscopy in patients with cirrhosis and recurrent HE [75]. Although this retrospective study only had 10 participants, it demonstrated that there was a sustained clinical response in six patients 20 weeks after treatment. These patients showed reductions in arterial ammonia concentration, Child–Pugh score, and model for end-stage liver disease score.

We recently completed a phase 1, double-blind, randomized clinical trial to study the effects of FMT enema on alcohol-use-disorder-related cirrhosis [76]. Although not designed to study FMT on outcomes of HE, this study demonstrated reduced alcohol craving, reduced urinary ethyglucuronide, improved cognition and psychosocial quality of life at day 15 compared to placebo. Moreover, there was a reduction in serum IL-6 and LPS binding protein and increased butyrate/isobutyrate compared to baseline in the FMT group. These metabolic changes are presumed to be due to the increased abundance of *Ruminococcaceae* in the treatment group. In regard to safety, there was a lower rate of serious adverse events in the FMT group compared to placebo.

Antibiotic resistance is a frequent complication of cirrhosis that leads to poor outcomes. FMT offers a promising therapy that may reduce the population of multidrug resistance organisms. We recently studied this by evaluating the expression of the antibiotic resistance gene (ARG) in patients with decompensated cirrhosis before and after healthy donor capsule and enema FMT [77]. There were 20 patients with cirrhosis in each trial (capsule and enema FMT), and all patients were on rifaximin, lactulose and proton pump inhibitors. ARGs were identified using metagenomics, and changes in ARG abundance were studied within and between groups. Expression of beta-lactamase was decreased post capsule FMT compared to baseline. Beta-lactamase, vancomycin and rifamycin ARGs were significantly lower at 4 weeks post-FMT compared to placebo. A reduction in rifamycin ARG in the interventional group was associated with cognitive improvement. In the enema FMT trial, beta-lactamase and vancomycin ARGs were decreased at day 7 post treatment compared to standard of care, and this reduction in ARGs persisted until day 15. These data suggest that ARG abundance is largely reduced after FMT in decompensated cirrhosis regardless of the route of administration.

## 7. Future Directions and Challenges

There are several ongoing and future trials studying several aspects of FMT including different routes of administration. The PROFIT trial is an ongoing study designed to assess the effect of FMT delivered directly into the small bowel of patients with cirrhosis [78]. In contrast to previous studies, the patients in this study are not to be pretreated with antibiotics. The idea is that direct instillation of FMT into the jejunum can directly target SIBO. This study is powered to study the feasibility and safety of this technique and not to assess clinical outcome. Preliminary data from this trial were recently presented at the 2020 digital international liver congress [79]. Twenty-one patients with confirmed cirrhosis were included, and 15 patients received FMT and six received placebo. Plasma ammonia was significantly reduced at day 30 compared to baseline. There was also a non-significant increase in plasma ammonia in the placebo group. Ammonia levels in stool were found to be increased in the placebo group but not in the FMT group.

A common criticism of the aforementioned studies is small sample sizes. This is certainly an issue, as small sample sizes can lead to inflated false discovery rate and inflated effect size estimation. Several large studies are ongoing and actively recruiting, one of which we are conducting [80]. This trial includes 100 participants with four groups randomized to oral and/or rectal FMT.

These early studies have shown promising results in treating HE with FMT. Several questions remain answered. One question is safety. Our studies have shown that FMT appears to be well tolerated and safe. A case report by DeFilipp et al. highlights the importance of donor screening to limit transmission of microorganisms [81]. They reported transmission of drug-resistant E. coli in two patients who had undergone FMT obtained from the same donor. Both patients developed bacteremia and one died. Other rare cases of bacteremia and death have been reported suggesting that FMT is not a benign procedure. Careful selection of donor and recipient must be done to minimize the risks. The FDA has updated their protocol on FMT in 2019 mandating that donors be screened for multi-drug-resistant organisms. However, since patients with cirrhosis have compromised intestinal membrane integrity and are immunocompromised, there is potential of transmission of other pathogenic organisms that are not routinely screened for.

The risk to benefit ratio of using FMT to treat chronic liver disease, cirrhosis and hepatic encephalopathy is yet to be established. The benefits are assuredly high, as there is no equivalent alternative therapy available to address the issue of dysbiosis in chronic liver disease. As mentioned previously, the risks include transmission of resistant organisms or other deleterious traits that are not yet measured. Furthermore, patients need to be accepting of this therapy, and it needs to be universally available. For this to occur, certain aspects regarding the implementation of FMT in clinical practice need to be established, particularly regarding the amount of donor material required, optimal route of administration, length and frequency of treatment, and interval between treatments. While FMT has been successful for the treatment of refractory *Clostridium difficile*, whereby gut microbiota is typically sterilized by antibiotics prior to replacement with a single inoculation of small amount of a donor’s sample, it may not be as simple in chronic liver disease. The dysbiosis in cirrhosis is complex and is not necessarily equivalent amongst patients, which makes its treatment selection challenging. Patients with cirrhosis are routinely treated with antibiotics for HE and spontaneous bacterial peritonitis, which further disturbs the microbiota. Dosing time is likely to be important, and preliminary data from the PROFIT trial, while small in number, have demonstrated this [82]. It is likely that multiple doses are required to achieve a meaningful therapeutic response. As mentioned previously, we have a large clinical trial with 100 participants to address the question of treatment mode of delivery and dose range (0–3, clinicaltrials.gov ID NCT03796598). Our study consists of four groups: dual oral and rectal FMT, oral FMT and rectal placebo, oral placebo and rectal FMT and oral and rectal placebo. Rectal-administered treatment is given once at day 2, and oral administered treatment is given at day 2 and day 30. Participants are then followed for 6 months, and the primary outcome of the study is to determine serious adverse events related to FMT. Secondary outcomes at 6 months include changes in microbial diversity in stool/blood/saliva, changes in intestinal permeability and change in HE status as determined by changes in EAS or PHES. To further assess treatment delivery, the PROFIT trial will be extending their study to recruit 300 more patients over two years to undergo multiple doses using a capsule delivery model in contrast to their previous endoscopically administered treatment. The possibility of long-lasting cure from FMT alone is highly unlikely given the multi-factorial nature of chronic liver disease. The complexity of chronic liver disease and cirrhosis require a multi-faceted treatment approach. It is clear that FMT is not intended to be used as a solo therapy and simply augments the existing armamentarium for treating liver disease.

The need for pre-procedure antibiotics for sterilization has not been studied extensively. Whether antibiotics need to be withheld post-FMT is not clear either, as this may not be feasible in many patients. Multiple routes of administration have been evaluated with many showing successful results; however, the superiority or non-inferiority of one route versus another is unknown. Utilizing FMT in clinical practice will only be possible once we find solutions to these questions.

## 8. Conclusions

Gut microbiota has been repeatedly demonstrated to play a major role in liver disease and its complications including HE. Many patients continue to have progressively worsening cirrhosis and more frequent and recurrent episodes of HE despite treatment. The gut microbiota represents an attractive therapeutic target. Early studies have shown encouraging findings with improvement in cognition and reduction in HE episodes following FMT, but larger studies are required and underway. There remains a need to standardize the treatment and explore the best route options along with integrating it with current therapies for HE.

## Figures and Tables

**Table 1:** Microbial changes in patients with cirrhosis.

Study	Population	Changes in Gut Microbiota	Additional Findings
Chen et al., 2011 [29]	24 Controls 36 Cirrhosis	Increased: Proteobacteria Decreased: Bacteroidetes	Child–Pugh score correlated positively with *Streptococcaceae* and negatively with *Lachnospiraceae*
Lu et al., 2011 [30]	32 Controls 31 Cirrhosis	Increased: *Enterobacteriaceae* Decreased: Firmicutes	Statistically significant decrease in *Bifidobacterium* to *Enterobacteriaceae* ratio in patients with decompensated hepatitis B cirrhosis.
Bajaj et al., 2012 [31]	10 Controls 25 Cirrhosis (17 with HE)	Increased: *Enterobacteriaceae*, *Leuconostocaceae*, *Lactobacillaceae*, *Alcaligenaceae*, *Fusobacteriaceae* Decreased: *Lachnospiraceae*, *Ruminococcaceae*, *Clostridiales XIV*	Specific bacterial families (*Alcaligenaceae*, *Enterobacteriaceae*, *Porphyromonadaceae*) were seen more commonly in patients with HE and were associated with alterations in cognition and inflammation.
Mutlu et al., 2012 [32]	18 Controls 28 Alcoholics without cirrhosis 19 Alcoholic with cirrhosis	Increased: Bacteroidetes Decreased: Proteobacteria	Dysbiosis seen in both alcoholic groups regardless of presence of cirrhosis.
Bajaj et al., 2012 [33]	17 Controls 60 Cirrhosis (24 with HE)	Increased: *Clostridium, Acidaminococcus, Enterococcus*, *Burkholderia*, *Ralstonia*, *Proteus* Decreased: *Dorea*, *Subdoligranulum*	Significant differences in mucosal microbiota between HE and patients without HE, reduction in *Roseburia* and increases in *Enterococcus*, *Veillonella*, *Megasphaera* and *Burkholderia.*
Zhang et al., 2013 [34]	26 with HE 25 Cirrhosis without HE	Increased: *Streptococcus salivarius* in HE	*Streptococcus salivarius* correlated negatively with cognitive function.
Kakiyama et al., 2013 [35]	14 Controls 47 Cirrhosis	Increased: *Enterobacteriaceae* Decreased: *Lachnospiraceae*, *Ruminococcaceae*, *Blautia*	Decreased fecal bile acids and reduced secondary bile acid conversion in patients with cirrhosis.
Bajaj et al., 2014 [36]	15 Controls 15 Cirrhosis	Decreased: *Lachnospiraceae*, *Ruminococcaceae*	Increased *Streptococcaceae* after PPI therapy in all groups.
Bajaj et al., 2014 [15]	25 Controls 219 Cirrhosis	Increased: *Enterococcaeae*, *Staphylococcaceae*, *Enterobacteriaceae* Decreased: *Ruminococcaceae*, *Lachnospiraceae*, *Veillonellaceae*, *Porphyromonadaceae*	Increase in *Enterobacteriaceae* after HE episode.
Qin et al., 2014 [16]	83 Controls 98 Cirrhosis	Increased: *Veillonella*, *Streptococcus*, *Clostridium* Decreased: Bacteroidetes	Significantly increased population of oral flora in cirrhosis.
Tuomisto et al., 2014 [37]	14 Controls 13 Cirrhosis	Increased: *Bacteroides* spp., *Enterobactericaeae*, *Enterobacter* spp.	Patients with alcoholic cirrhosis had increased amounts of enterobacteria in feces.
Bajaj et al., 2015 [38]	32 Controls 102 Cirrhosis	Increased: *Enterobacteriaceae*, *Enterococcaceae* Decreased: *Lachnospiraceae*, *Ruminococcaceae*, *Clostridiaceae*	Patients with cirrhosis had dybiosis in saliva and stool.
Bajaj et al., 2015 [39]	94 Controls 278 Cirrhosis	Increased: *Lactobacillaceae*, *Enterococcaceae*, *Enterobacteriaceae*, *Pasteurellaceae* Decreased: *Bacteroidaceae*, *Porphyromonadaceae*, *Clostridiales XIV*, *Lachnospiraceae*, *Ruminococcaceae*	Patients with cirrhosis and diabetes were found to have increased *Bacteroidaceae* and reduced *Ruminococcaceae* in stool.
Chen et al., 2015 [40]	50 Controls 79 Cirrhosis	Increased: *Pasteurellaceae*, *Streptococcaceae*, *Enterococcaceae* Decreased: *Bacteroidaceae*, *Ruminococcaceae*, *Lachnospiraceae*	Individuals who developed HE had reduced population of *Lachnospiraceae*.
Ahluwalia et al., 2016 [41]	40 Controls 147 Cirrhosis	Increased: *Lactobacillaceae*, *Enterococcaceae*, *Clostridiales XIV*, *Lachnospiraceae*, *Enterobacteriaceae*	Patients with cirrhosis and HE had increased *Staphylococcaceae*, *Enterococcaceae*, *Porphyromonadaceae*, *Lactobacillaceae*
Chen et al., 2016 [42]	28 Controls 30 Cirrhosis	Increased: *Veillonella*, *Megasphaera*, *Dialister*, *Atopobium*, *Prevotella*	PPI reduced *Cloacibacterium* and increased *Dialister*
Santiago et al., 2016 [43]	17 Controls 60 Cirrhosis	Decreased: *Clostridiales*, *Roseburia faecis*, *Alistipes putredinis*, *Oscillospira*, *Mogibacteriaceae*, *Dehalobacterium*	Patients with ascites had elevation in markers of serum microbial translocation. Fecal microbiome composition was more altered in patients with ascites compared to those without.
Dubinkina et al., 2017 [44]	72 Alcoholics 27 Alcoholic cirrhosis	Increased: *Streptococcus constellatus*, *Streptococcus salivarius*, *Veillonella atypica*, *Veillonella dispar*, and *Veillonella parvula* Decreased: *Parabacteroides*	Increased abundance of oral microbes in patients with cirrhosis.
Sung et al., 2019 [45]	13 Controls 97 Cirrhosis (62 with HE)	Increased: *Veillonella parvula*, *Clostridium* XI, *Prevotella*, *Enterococcus*, *Schlegelella*, *Megasphaeae*, *Lactobacillus* Decreased: *Phascolarctobacterium*, *Bacteroides*, *Alistipes*	Increased abundance of *Alistipes*, *Bacteroides* and *Phascolarctobacterium* associated with HE recurrence.

## Data Availability

Not applicable to this study.

## References

[1] Vilstrup H.V.A., Amodio P., Bajaj J., Cordoba J., Ferenci P., Mullen K.D., Weissenborn K., Wong P. (2014). Hepatic encephalopathy in chronic liver disease: 2014 Practice Guideline by the American Association for the Study of Liver Diseases and the European Association for the Study of the Liver. Hepatology.

[2] Patidar K.R., Bajaj J.S. (2015). Covert and Overt Hepatic Encephalopathy: Diagnosis and Management. Clin. Gastroenterol. Hepatol..

[3] Neff G., Zachry W. (2018). Systematic Review of the Economic Burden of Overt Hepatic Encephalopathy and Pharmacoeconomic Impact of Rifaximin. Pharmacoeconomics.

[4] Butterworth R.F. (2019). Hepatic Encephalopathy in Cirrhosis: Pathology and Pathophysiology. Drugs.

[5] Ochoa-Sanchez R., Rose C.F. (2018). Pathogenesis of Hepatic Encephalopathy in Chronic Liver Disease. J. Clin. Exp. Hepatol..

[6] Bajaj J.S. (2014). The role of microbiota in hepatic encephalopathy. Gut Microbes..

[7] Garcovich M., Zocco M.A., Roccarina D., Ponziani F.R., Gasbarrini A. (2012). Prevention and treatment of hepatic encephalopathy: Focusing on gut microbiota. World J. Gastroenterol..

[8] Rai R., Saraswat V.A., Dhiman R.K. (2015). Gut microbiota: Its role in hepatic encephalopathy. J. Clin. Exp. Hepatol..

[9] Nardelli S., Ridola L., Gioia S., Riggio O. (2018). Management of Hepatic Encephalopathy Not Responsive to First-Line Treatments. Curr. Treat. Options Gastroenterol..

[10] Guarner F., Malagelada J.R. (2003). Gut flora in health and disease. Lancet.

[11] Lynch S.V., Pedersen O. (2016). The Human Intestinal Microbiome in Health and Disease. N. Engl. J. Med..

[12] O’Keefe S.J. (2008). Nutrition and colonic health: The critical role of the microbiota. Curr. Opin. Gastroenterol..

[13] Eckburg P.B., Bik E.M., Bernstein C.N., Purdom E., Dethlefsen L., Sargent M., Gill S.R., Nelson K.E., Relman D.A. (2005). Diversity of the human intestinal microbial flora. Science.

[14] Baquero F., Nombela C. (2012). The microbiome as a human organ. Clin. Microbiol. Infect..

[15] Bajaj J.S., Heuman D.M., Hylemon P.B., Sanyal A.J., White M.B., Monteith P., Noble N.A., Unser A.B., Daita K., Fisher A.R. (2014). Altered profile of human gut microbiome is associated with cirrhosis and its complications. J. Hepatol..

[16] Qin N., Yang F., Li A., Prifti E., Chen Y., Shao L., Guo J., Le Chatelier E., Yao J., Wu L. (2014). Alterations of the human gut microbiome in liver cirrhosis. Nature.

[17] Peng L., Li Z.-R., Green R.S., Holzman I.R., Lin J.L. (2009). Butyrate enhances the intestinal barrier by facilitating tight junction assembly via activation of AMP-activated protein kinase in Caco-2 cell monolayers. J. Nutr..

[18] Ríos-Covián D., Ruas-Madiedo P., Margolles A., Gueimonde M., Reyes-Gavilán C.G.D.L., Salazar N. (2016). Intestinal Short Chain Fatty Acids and their Link with Diet and Human Health. Front. Microbiol..

[19] Ridlon J.M., Kang D.J., Hylemon P.B. (2006). Bile salt biotransformations by human intestinal bacteria. J. Lipid Res..

[20] Begley M., Gahan C.G., Hill C. (2005). The interaction between bacteria and bile. FEMS Microbiol. Rev..

[21] Vlahcevic Z.R., Buhac I., Bell C.C., Swell L. (1970). Abnormal metabolism of secondary bile acids in patients with cirrhosis. Gut.

[22] Swann J.R., Want E.J., Geier F.M., Spagou K., Wilson I.D., Sidaway J.E., Nicholson J.K., Holmes E. (2011). Systemic gut microbial modulation of bile acid metabolism in host tissue compartments. Proc. Natl. Acad. Sci. USA.

[23] Tranah T.H., Edwards L.A., Schnabl B., Shawcross D.L. (2020). Targeting the gut-liver-immune axis to treat cirrhosis. Gut.

[24] Wigg A.J., Roberts-Thomson I.C., Dymock R.B., McCarthy P.J., Grose R.H., Cummins A.G. (2001). The role of small intestinal bacterial overgrowth, intestinal permeability, endotoxaemia, and tumour necrosis factor alpha in the pathogenesis of non-alcoholic steatohepatitis. Gut.

[25] Pande C., Kumar A., Sarin S.K. (2009). Small-intestinal bacterial overgrowth in cirrhosis is related to the severity of liver disease. Aliment. Pharmacol. Ther..

[26] Bauer T.M., Steinbrückner B., Brinkmann F.E., Ditzen A.K., Schwacha H., Aponte J.J., Pelz K., Kist M., Blum H.E. (2001). Small intestinal bacterial overgrowth in patients with cirrhosis: Prevalence and relation with spontaneous bacterial peritonitis. Am. J. Gastroenterol..

[27] Betrapally N.S., Gillevet P.M., Bajaj J.S. (2017). Gut microbiome and liver disease. Transl. Res..

[28] Gómez-Hurtado I., Such J., Francés R. (2016). Microbiome and bacterial translocation in cirrhosis. Microbioma y traslocación bacteriana en la cirrosis. Gastroenterol. Hepatol..

[29] Chen Y., Yang F., Lu H., Wang B., Chen Y., Lei D., Wang Y., Zhu B., Li L. (2011). Characterization of fecal microbial communities in patients with liver cirrhosis. Hepatology.

[30] Lu H., Wu Z., Xu W., Yang J., Chen Y., Li L. (2011). Intestinal microbiota was assessed in cirrhotic patients with hepatitis B virus infection. Intestinal microbiota of HBV cirrhotic patients. Microb. Ecol..

[31] Bajaj J.S., Ridlon J.M., Hylemon P.B., Thacker L.R., Heuman D.M., Smith S., Sikaroodi M., Gillevet P.M. (2012). Linkage of gut microbiome with cognition in hepatic encephalopathy. Am. J. Physiol. Liver Physiol..

[32] Mutlu E.A., Gillevet P.M., Rangwala H., Sikaroodi M., Naqvi A., Engen P.A., Kwasny M., Lau C.K., Keshavarzian A. (2012). Colonic microbiome is altered in alcoholism. Am. J. Physiol. Liver Physiol..

[33] Bajaj J.S., Hylemon P.B., Ridlon J.M., Heuman D.M., Daita K., White M.B., Monteith P., Noble N.A., Sikaroodi M., Gillevet P.M. (2012). Colonic mucosal microbiome differs from stool microbiome in cirrhosis and hepatic encephalopathy and is linked to cognition and inflammation. Am. J. Physiol. Liver Physiol..

[34] Zhang Z., Zhai H., Geng J., Yu R., Ren H., Fan H., Shi P. (2013). Large-scale survey of gut microbiota associated with MHE Via 16S rRNA-based pyrosequencing. Am. J. Gastroenterol..

[35] Kakiyama G., Pandak W.M., Gillevet P.M., Hylemon P.B., Heuman D.M., Daita K., Takei H., Muto A., Nittono H., Ridlon J.M. (2013). Modulation of the fecal bile acid profile by gut microbiota in cirrhosis. J. Hepatol..

[36] Bajaj J.S., Cox I.J., Betrapally N.S., Heuman D.M., Schubert M.L., Ratneswaran M., Hylemon P.B., White M.B., Daita K., Noble N.A. (2014). Systems biology analysis of omeprazole therapy in cirrhosis demonstrates significant shifts in gut microbiota composition and function. Am. J. Physiol. Liver Physiol..

[37] Tuomisto S., Pessi T., Collin P., Vuento R., Aittoniemi J., Karhunen P.J. (2014). Changes in gut bacterial populations and their translocation into liver and ascites in alcoholic liver cirrhotics. BMC Gastroenterol..

[38] Bajaj J.S., Betrapally N.S., Hylemon P.B., Heuman D.M., Daita K., White M.B., Unser A., Thacker L.R., Sanyal A.J., Kang D.J. (2015). Salivary microbiota reflects changes in gut microbiota in cirrhosis with hepatic encephalopathy. Hepatology.

[39] Bajaj J.S., Betrapally N.S., Hylemon P.B., Thacker L.R., Daita K., Kang D.J., White M.B., Unser A.B., Fagan A., Gavis E.A. (2015). Gut Microbiota Alterations can predict Hospitalizations in Cirrhosis Independent of Diabetes Mellitus. Sci. Rep..

[40] Chen Y., Guo J., Qian G., Fang D., Shi D., Guo L., Li L. (2015). Gut dysbiosis in acute-on-chronic liver failure and its predictive value for mortality. J. Gastroenterol. Hepatol..

[41] Ahluwalia V., Betrapally N.S., Hylemon P.B., White M.B., Gillevet P.M., Unser A.B., Fagan A., Daita K., Heuman D.M., Zhou H. (2016). Impaired Gut-Liver-Brain Axis in Patients with Cirrhosis. Sci. Rep..

[42] Chen Y., Ji F., Guo J., Shi D., Fang D., Li L. (2016). Dysbiosis of small intestinal microbiota in liver cirrhosis and its association with etiology. Sci. Rep..

[43] Santiago A., Pozuelo M., Poca M., Gely C., Nieto J.C., Torras X., Román E., Campos D., Sarrabayrouse G., Vidal S. (2016). Alteration of the serum microbiome composition in cirrhotic patients with ascites. Sci. Rep..

[44] Dubinkina V.B., Tyakht A., Odintsova V.Y., Yarygin K.S., Kovarsky B.A., Fedorov D.E., Ischenko D., Popenko A.S., Alekseev D., Taraskina A.E. (2017). Links of gut microbiota composition with alcohol dependence syndrome and alcoholic liver disease. Microbiome.

[45] Sung C.M., Lin Y.-F., Chen K.-F., Ke H.-M., Huang H.-Y., Gong Y.-N., Tsai W.-S., You J.-F., Lu M.J., Cheng H.-T. (2019). Predicting Clinical Outcomes of Cirrhosis Patients With Hepatic Encephalopathy From the Fecal Microbiome. Cell. Mol. Gastroenterol. Hepatol..

[46] Bajaj J.S., Kakiyama G., Cox I.J., Nittono H., Takei H., White M., Fagan A., Gavis E.A., Heuman D.M., Gilles H.C. (2018). Alterations in gut microbial function following liver transplant. Liver Transpl..

[47] Schoeler M., Caesar R. (2019). Dietary lipids, gut microbiota and lipid metabolism. Rev. Endocr. Metab. Disord..

[48] Zhou D., Fan J.G. (2019). Microbial metabolites in non-alcoholic fatty liver disease. World J. Gastroenterol..

[49] Kiouptsi K., Ruf W., Reinhardt C. (2018). Microbiota-Derived Trimethylamine. Circ. Res..

[50] Hemming A.W., Gallinger S., Greig P.D., Cattral M.S., Langer B., Taylor B.R., Verjee Z., Giesbrecht E., Nakamachi Y., Furuya K.N. (2001). The hippurate ratio as an indicator of functional hepatic reserve for resection of hepatocellular carcinoma in cirrhotic patients. J. Gastrointest. Surg..

[51] Brial F., Le Lay A., Dumas M.E., Gauguier D. (2018). Implication of gut microbiota metabolites in cardiovascular and metabolic diseases. Cell. Mol. Life Sci..

[52] Shah A., Shanahan E., Macdonald G.A., Fletcher L., Ghasemi P., Morrison M., Jones M.P., Holtmann G.J. (2017). Systematic Review and Meta-Analysis: Prevalence of Small Intestinal Bacterial Overgrowth in Chronic Liver Disease. Semin. Liver Dis..

[53] Fukui H. (2015). Gut Microbiota and Host Reaction in Liver Diseases. Microorganisms.

[54] Adike A., DiBaise J.K. (2018). Small Intestinal Bacterial Overgrowth: Nutritional Implications, Diagnosis, and Management. Gastroenterol. Clin. N. Am..

[55] Rezaie A., Pimentel M., Rao S.S. (2016). How to Test and Treat Small Intestinal Bacterial Overgrowth: An Evidence-Based Approach. Curr. Gastroenterol. Rep..

[56] Ponziani F.R., Gerardi V., Gasbarrini A. (2016). Diagnosis and treatment of small intestinal bacterial overgrowth. Expert Rev. Gastroenterol. Hepatol..

[57] Quigley E.M.M. (2019). The Spectrum of Small Intestinal Bacterial Overgrowth (SIBO). Curr. Gastroenterol. Rep..

[58] Maslennikov R., Pavlov C., Ivashkin V. (2018). Small intestinal bacterial overgrowth in cirrhosis: Systematic review and meta-analysis. Hepatol. Int..

[59] Gupta A., Dhiman R.K., Kumari S., Rana S., Agarwal R., Duseja A., Chawla Y. (2010). Role of small intestinal bacterial overgrowth and delayed gastrointestinal transit time in cirrhotic patients with minimal hepatic encephalopathy. J. Hepatol..

[60] Lunia M.K., Sharma B.C., Sachdeva S. (2013). Small intestinal bacterial overgrowth and delayed orocecal transit time in patients with cirrhosis and low-grade hepatic encephalopathy. Hepatol. Int..

[61] Zhang Y., Feng Y., Cao B., Tian Q. (2016). The effect of small intestinal bacterial overgrowth on minimal hepatic encephalopathy in patients with cirrhosis. Arch. Med. Sci..

[62] Fukui H., Wiest R. (2016). Changes of Intestinal Functions in Liver Cirrhosis. Inflamm. Intest. Dis..

[63] Revaiah P.C., Kochhar R., Rana S.V., Berry N., Ashat M., Dhaka N., Reddy Y.R., Sinha S.K. (2018). Risk of small intestinal bacterial overgrowth in patients receiving proton pump inhibitors *versus* proton pump inhibitors plus prokinetics. JGH Open.

[64] Singh R.K., Chang H.-W., Yan D., Lee K.M., Ucmak D., Wong K., Abrouk M., Farahnik B., Nakamura M., Zhu T.H. (2017). Influence of diet on the gut microbiome and implications for human health. J. Transl. Med..

[65] Bajaj J.S., Idilman R., Mabudian L., Hood M., Fagan A., Turan D., White M.B., Karakaya F., Wang J., Atalay R. (2018). Diet affects gut microbiota and modulates hospitalization risk differentially in an international cirrhosis cohort. Hepatology.

[66] Ritchie M.L., Romanuk T.N. (2012). A meta-analysis of probiotic efficacy for gastrointestinal diseases. PLoS ONE.

[67] McLoughlin R.F., Berthon B.S., Jensen M.E., Baines K.J., Wood L.G. (2017). Short-chain fatty acids, prebiotics, synbiotics, and systemic inflammation: A systematic review and meta-analysis. Am. J. Clin. Nutr..

[68] Saab S., Suraweera D., Au J., Saab E.G., Alper T.S., Tong M.J. (2016). Probiotics are helpful in hepatic encephalopathy: A meta-analysis of randomized trials. Liver Int..

[69] Brandt L.J. (2012). Fecal transplantation for the treatment of Clostridium difficile infection. Gastroenterol. Hepatol. N. Y..

[70] Borody T.J., Clancy A. (2019). Fecal microbiota transplantation for ulcerative colitis-where to from here?. Transl. Gastroenterol. Hepatol..

[71] Kao D., Roach B., Park H., Hotte N., Madsen K., Bain V., Tandon P. (2016). Fecal microbiota transplantation in the management of hepatic encephalopathy. Hepatology.

[72] Bajaj J.S., Kassam Z., Fagan A., Gavis E.A., Liu E., Cox I.J., Kheradman R., Heuman D., Wang J., Gurry T. (2017). Fecal microbiota transplant from a rational stool donor improves hepatic encephalopathy: A randomized clinical trial. Hepatology.

[73] Bajaj J.S., Fagan A., Gavis E.A., Kassam Z., Sikaroodi M., Gillevet P.M. (2019). Long-term Outcomes of Fecal Microbiota Transplantation in Patients with Cirrhosis. Gastroenterology.

[74] Bajaj J.S., Salzman N.H., Acharya C., Sterling R.K., White M.B., Gavis E.A., Fagan A., Hayward M., Holtz M.L., Matherly S. (2019). Fecal Microbial Transplant Capsules Are Safe in Hepatic Encephalopathy: A Phase 1, Randomized, Placebo-Controlled Trial. Hepatology.

[75] Mehta R., Kabrawala M., Nandwani S., Kalra P., Patel C., Desai P., Parekh K. (2018). Preliminary experience with single fecal microbiota transplant for treatment of recurrent overt hepatic encephalopathy-A case series. Indian J. Gastroenterol..

[76] Bajaj J.S., Gavis E.A., Fagan A., Wade J.B., Thacker L.R., Fuchs M., Patel S., Davis B.C., Meador J., Puri P. (2020). A Randomized Clinical Trial of Fecal Microbiota Transplant for Alcohol Use Disorder. Hepatology.

[77] Bajaj J.S., Shamsaddini A., Fagan A., Sterling R.K., Gavis E., Khoruts A., Fuchs M., Lee H., Sikaroodi M., Gillevet P.M. (2020). Fecal Microbiota Transplant in Cirrhosis Reduces Gut Microbial Antibiotic Resistance Genes: Analysis of Two Trials. Hepatol. Commun..

[78] Woodhouse C.A., Patel V.C., Goldenberg S., Sanchez-Fueyo A., China L., O’Brien A., Flach C., Douiri A., Shawcross D. (2019). PROFIT, a PROspective, randomised placebo controlled feasibility trial of Faecal mIcrobiota Transplantation in cirrhosis: Study protocol for a single-blinded trial. BMJ Open.

[79] Woodhouse C., Edwards L., Mullish B.H., Kronsten V., Tranah T., Zamalloa A., Flach C., Douiri A., Marchesi J., Patel V. (2019). Results of the PROFIT trial, a PROspective randomized placebo-controlled feasibility trial of Faecal mIcrobiota Transplantation in advanced cirrhosis. BMJ Open.

[80] Hatton G.B., Ran S., Tranah T.H., Shawcross D.L. (2020). Lessons Learned from Faecal Microbiota Transplantation in Cirrhosis. Curr Hepatol. Rep..

[81] DeFilipp Z., Bloom P.P., Soto M.T., Mansour M.K., Sater M.R., Huntley M.H., Turbett S., Chung R.T., Chen Y.-B., Hohmann E.L. (2019). Drug-Resistant *E. coli* Bacteremia Transmitted by Fecal Microbiota Transplant. N. Engl. J. Med..

[82] Edwards L., Woodhouse C., Kronsten V., Zamalloa A., Tranah T., Patel V., Sanchez-Fueyo A., Goldenberg S., Shawcross D.L. (2020). Faecal microbiota transplantation reduces pathogenic burden and is anti-inflammatory in patients with advanced cirrhosis. J. Hepatol..

